# Complete chloroplast-based analysis reveals the emergence of candidate new species and revised divergence time in *Vincetoxicum*

**DOI:** 10.1007/s13258-026-01808-1

**Published:** 2026-09-28

**Authors:** Bumjin Jang, Byung Jun Jin, Yun Ji Park, Sumin Jeong, Yeseul Kim, Thi Tuyen Cu, Bo-Mi Nam, Inkyu Park

**Affiliations:** 1 Department of Biology, Changwon National University, Changwon, 51140 Republic of Korea; 2 Global Advanced Institute of Nano Technology, Changwon National University, Changwon, 51140 Republic of Korea; 3 Division of Botany, Honam National Institute of Biological Resources, Mokpo, 58762 Republic of Korea

**Keywords:** *Vincetoxicum inamoenum*, *Vincetoxicum* sp., Chloroplast genome, Comparative analysis, Phylogenetic tree, Divergence time estimate

## Abstract

**Background:**

The taxonomic understanding of *Vincetoxicum* remains unresolved due to the lack of genetic information for *Vincetoxicum inamoenum*. *Vincetoxicum* sp. was recently identified as a morphologically distinct putative new species related to *V. inamoenum*, increasing the necessity for a more accurate reconstruction of the taxonomic history of *Vincetoxicum* based on molecular and phylogenetic evidence from *V. inamoenum* and *V.* sp.

**Objective:**

To assemble the complete chloroplast genomes of *V*. *inamoenum* and *V*. sp., elucidate their genetic divergence from 12 *Vincetoxicum* species, and determine the phylogenetic relationship and divergence time of the candidate new species of *Vincetoxicum*.

**Methods:**

Complete chloroplast genomes of *V. inamoenum* and *V*. sp. were assembled. Repeat sequences, nucleotide diversity, and selective pressure between *V. inamoenum* and *V*. sp. were analyzed using 12 previously reported *Vincetoxicum* species. Phylogenetic analysis was performed to determine the phylogenetic position and divergence time.

**Results:**

The chloroplast genomes of *V. inamoenum* and *V*. sp. were 160,787 and 160,849 base pairs long, respectively. Highly variable regions were identified between the two *Vincetoxicum* species (*accD-psaI*) and among the 14 *Vincetoxicum* species (*trnH-psbA*). The *psaI*,* rpl33* and *ycf2* genes exhibited positive selection. The phylogenetic tree showed that *V. inamoenum* and *V*. sp. formed a sister group and reconstructed the phylogenetic relationships within *Vincetoxicum*. Divergence time estimation indicated that *V. inamoenum* and *V.* sp. diverged approximately 1.182 million years ago (95% Highest Posterior Density: 0.181–2.751).

**Conclusions:**

Distinct genetic differences and divergence time indicate that *V*. sp. is a putative new species and provide a more comprehensive and accurate understanding of the genomic diversity and phylogenetic insight into *Vincetoxicum*.

**Supplementary Information:**

The online version contains supplementary material available at https://doi.org/10.1007/s13258-026-01808-1.

## Introduction

The genus *Vincetoxicum* Wolf. belongs to the family Apocynaceae, subfamily Asclepiadoideae, tribe Asclepiadeae, and subtribe Tylophorinae. *Vincetoxicum* is widely distributed across Africa, Europe, Australia, and Asia, including the Far East, and is an invasive species in North America (Ernst and Cappuccino 2005; Averill et al. 2011; DiTommaso et al. 2018; Shah et al. 2021; Mochizuki et al. 2024; Prameela and Krishnasamy 2024; Zhang and Hu 2024). Within the genus, *Vincetoxicum inamoenum* Maxim. is a perennial herb distributed in East Asian countries, including China, Japan, and Korea, and parts of the Russian Far East (e.g., Khabarovsk, Primorye, and Sakhalin).

*Vincetoxicum* spp. are widely used medicinal plants (Kara et al. 2018; Westbrook et al. 2023; Prameela and Krishnasamy 2024). In Korea and China, the roots of *V. atratum* and *V. versicolor* are used as herbal medicines in Cynanchi Atrati Radix Et Rhizoma (Yu et al. 2021; Lee et al. 2022; Ren et al. 2025). In addition, *Vincetoxicum* species are recognized for exhibiting various therapeutic activities (anticancer, antimicrobial, anti-inflammatory, antispasmodic, and antimalarial activities), and research on these properties has been conducted (Prameela and Krishnasamy 2024). *V. inamoenum* is traditionally used to treat postpartum depression, pregnancy enuresis, scabies, and lymphadenitis (Han et al. 2018). For these reasons, *Vincetoxicum* is an important genus both economically and pharmacologically.

In Korean taxonomic studies of Asclepiadoideae, some populations of *V. inamoenum* exhibited distinct morphological differences, which were initially regarded as intraspecific variation, but were later suggested to represent a putative new species (Nam 2017). In the present study, *V. inamoenum* was clearly distinguished by its short peduncles, a corona longer than the gynostegium with an incurved apex, and pollinia with a punctate surface. In contrast, the putative new species has long peduncles, a corona nearly equal in length to the gynostegium, a blunt apex, and pollinia with a reticulate surface (Nam 2017). In addition, phylogenetic analysis of the chloroplast regions (*matK*, *trnL* intron, *trnL-F* and *psbA-trnH*) revealed a separate topology between *V. inamoenum* and the putative new species (Nam 2017). However, morphology-based taxonomy has been constrained by intraspecific variation and phenotypic plasticity, leading to inconsistencies in molecular results (Haider 2011; Ko and Jeun 2025). Therefore, species identification and classification rely increasingly on an integrative framework that combines morphology with molecular, biogeographical, phylogenetic, and chemotaxonomic data to reach robust conclusions (Kim 1996; Maltsev and Erst 2023).

Chloroplast genomes have advantages in evolution and phylogenetic inference because of their uniparental inheritance, low frequency of recombination, low nucleotide substitution rates, and conserved gene content and order (Li et al. 2013; Ren et al. 2020; Sun et al. 2022; Bausher et al. 2006; Xue et al. 2019; Henriquez et al. 2020; Lu et al. 2022; Zhang et al. 2022a, b; Duan et al. 2024). Many previous studies have demonstrated that phylogenetic analyses using whole-chloroplast datasets provide higher support values and contribute to a clearer resolution of phylogenetic relationships (Feng et al. 2020; Kyalo et al. 2020; Xiong et al. 2023). In addition, non-coding regions of chloroplasts, which have a relatively high substitution rate and more rapid evolutionary events than coding regions, are useful for distinguishing closely related taxa at the interspecific and subspecific levels (Downie et al. 2000; Huang et al. 2022).

Although the phylogenetic position of *V. inamoenum* has already been established, previous analyses were restricted to limited genetic regions. Molecular evidence clarifying the phylogenetic relationship between *V. inamoenum* and the newly proposed species, as well as supporting its taxonomic recognition, is almost absent. Therefore, a comparative phylogenetic analysis based on complete chloroplast genomes is required to provide robust evidence for the putative new species and reassess the phylogenetic position of *V. inamoenum.* However, complete chloroplast genome data for *V. inamoenum* and the putative new species are absent. To-date, at least 13 complete *Vincetoxicum* chloroplast genomes are available in the NCBI database (Rao et al. 2018; Guan and Zhang 2019; Xiong et al. 2019; Zhang and Zhang 2019; Yu et al. 2021; Ye et al. 2022; Liu et al. 2023; Ren et al. 2025; Han et al. 2026). Studies on *V. versicolor*,* V. mongolicum*, and *V. atratum* have provided insights into their genetic diversity through comparative analyses of chloroplast genomes, and research on *V. versicolor* has been extended to transcriptome analyses (Yu et al. 2021; Liu et al. 2023; Ren et al. 2025). To the best of our knowledge, comprehensive selective pressure analyses and fine-scale divergence time estimates among species within *Vincetoxicum* remain lacking. Previous divergence time analyses, including *those of Vincetoxicum* have primarily focused on broad-scale aspects, such as intercontinental lineage dispersal, evolutionary divergence among genera at the family level, and taxonomic integration with closely related genera (Liede-Schumann et al. 2012, 2016; Fishbein et al. 2018). Consequently, the lack of selective pressure and interspecific divergence time estimation analyses makes it difficult to obtain comprehensive evolutionary insights into the genus *Vincetoxicum*.

In this study, we analyzed a morphologically distinct population of *V. inamoenum* by assembling and comparing the complete chloroplast genomes of *V.* sp. (GenBank accession no. PX975501), and *V. inamoenum* (GenBank accession no. PX975500) to assess their genetic divergence and to provide molecular data for taxonomic distinction. In addition, by integrating the two newly assembled chloroplast genomes with 12 previously published complete *Vincetoxicum* chloroplast genomes (14 species in total), we conducted comparative genomic analyses to quantify the overall genetic diversity within the genus. Furthermore, we performed an expanded phylogenetic analysis to clarify the molecular phylogenetic relationships within *Vincetoxicum* with greater precision than previous studies. Through these objectives, we aimed to provide molecular evidence of this new species and further advance the genomic understanding of the genus *Vincetoxicum*.

## Materials and methods

### Plant materials

**Fig. 1:**
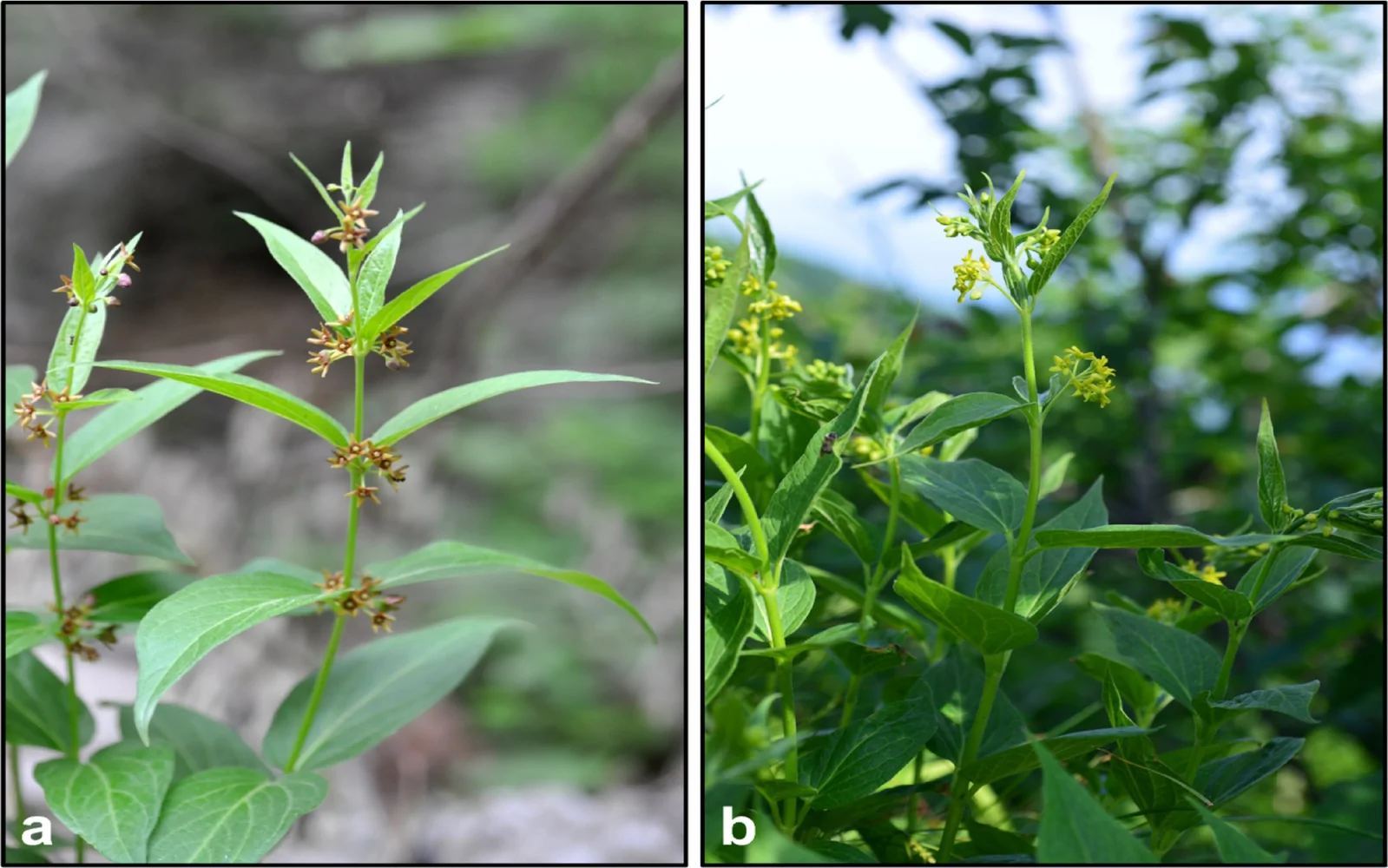
Habit photographs of two *Vincetoxicum* species. **a***Vincetoxicum inamoenum*, **b***Vincetoxicum* sp.

Fresh leaf tissues of *V. inamoenum* and *V.* sp. were collected directly from Deokpung Valley, Samcheok-si, Gangwon-do, South Korea (N37°06′35″ E129°10′50″) and Mt. Deogyusan, Muju-gun, Jeollabuk-do, South Korea (N35°51′35″ E127°44′49″) (Fig. 1). For genomic DNA extraction, dried samples of each species were kindly provided by Bo-Mi Nam (Division of Botany, Honam National Institute of Biological Resources, Mokpo, South Korea).

### Genome sequencing and assembly

Each sample stored at − 80 ℃ was homogenized using a mortar and pestle, and then genomic DNA was extracted by the cetyltrimethylammonium bromide (CTAB) method of Doyle et al. (1990) with slight modifications. Genomic DNA validation for concentration and purity was performed using a BioDrop µLite+ Microvolume Spectrophotometer (Harvard Bioscience, Inc., Holliston, MA, USA). A genomic DNA library was prepared using a TruSeq Nano DNA Library Preparation Kit (Illumina Inc., San Diego, CA, USA) according to the manufacturer’s instructions. Paired-end sequencing [2 × 150 base pairs (bp)] was performed using an Illumina MiSeq platform. To ensure data quality, raw reads were trimmed using Trimmomatic ver. 0.39 (Bolger et al. 2014) with a Phred quality score threshold of ≥ 20, and only high-quality reads (clean reads) were retained for subsequent analyses. These quality-filtered reads were then used for *de novo* assembly of the *Vincetoxicum* chloroplast genome using GetOrganelle ver. 1.7.7.0 (Jin et al. 2020) with default parameter settings. The assembled contigs corresponding to the plastid genome were identified by alignment with known reference sequences using NUCmer in the MUMmer package (Delcher et al. 2003). To validate and orient these contigs, they were aligned to the complete chloroplast genome of *Cynanchum wilfordii* (GenBank accession number NC_029459). SOAPdenovo GapCloser (Luo et al. 2012) was used to fill the remaining sequence gaps and enhance the overall continuity and completeness of the assembly. Finally, to verify the correctness of the assembly, the assembled *Vincetoxicum* chloroplast genomes were mapped to chloroplast data from *C. wilfordii* using the Burrows-Wheeler Aligner (BWA) (Li 2013) and sequence alignment map (SAM) tools (Li et al. 2009).

### Genome annotation and codon usage pattern

Gene structures, including protein-coding genes, rRNAs, and tRNAs, in the assembled chloroplast genomes of the two *Vincetoxicum* species were separately annotated using the *C. wilfordii* genome (NC_029459) as the common reference genome with two web-based annotation tools, GeSeq (Tillich et al. 2017; for annotation of protein-coding genes and rRNAs) and tRNAscan-SE ver. 2.0.7 (Chan and Lowe 2019; for annotation of tRNAs). Inverted repeat (IR) regions were identified using Repeat Finder ver. 1.0.1 plugin in Geneious Prime (Kearse et al. 2012). The final annotation refinement step was conducted manually using Geneious software (Kearse et al. 2012). Based on the chloroplast genome annotations, a circular genome map was generated using OGDRAW ver. 1.3.1 (Greiner et al. 2019) to visualize chloroplast genome structure and content. Codon usage patterns, including amino acid numbers and relative synonymous codon usage (RSCU) values, were analyzed by comparing 88 CDSs extracted from each of the two *Vincetoxicum* genomes using MEGA 11 (Tamura et al. 2021). An RSCU value greater than 1 indicates a predominantly used (or preferred) codon, whereas a value less than 1 indicates a less frequently used (or non-preferred) codon. Codon usage data were grouped according to the corresponding amino acids, with RSCU values and the number of amino acids calculated accordingly.

### Repeat analysis

Repeat analysis was conducted to identify repeat sequences in 14 *Vincetoxicum* chloroplast genomes. Simple sequence repeats (SSRs) were analyzed using the MISA-web tool (Beier et al. 2017) with the following minimum repeat thresholds: 10 mononucleotides, six dinucleotides, five trinucleotide, four tetranucleotide, and three pentanucleotide and hexanucleotide repeats. Tandem repeats were identified using Tandem Repeats Finder (TRF) (Benson 1999), with a minimum alignment score of 50, a maximum period size of 500, and a repeat identity threshold of ≥ 90%. Overlapping repeats were excluded from analysis. Repeat types, including forward, reverse, complement, and palindromic repeats, were identified using REPuter (Kurtz et al. 2001) with the following parameters: a Hamming distance of three and a minimum repeat size of 30 bp.

### Comparative analysis

For comparative analysis of chloroplast genomes among 14 *Vincetoxicum* species, IR expansion and contraction were identified using Geneious software by comparison with *C. wilfordii* (Kearse et al. 2012). To compare and visualize sequence variation among *Vincetoxicum* species, sequence alignment was carried out using the Shuffle-LAGAN mode in the mVISTA program (Frazer et al. 2004) with *V. inamoenum* as the reference. Nucleotide diversity (Pi) values were calculated using DnaSP ver. 6 (Rozas et al. 2017), with regions ≥ 200 bp included in the analysis. To assess the overall nucleotide diversity of the *Vincetoxicum* species, Pi value analyses were conducted using four species groupings: [1] 14 *Vincetoxicum* species; [2] *V. inamoenum* and *V.* sp., assembled in this study; [3] the 9 species comprising Group A; and [4] the 5 species comprising Group B. Groups A and B were identified as two major groups in this phylogenetic analysis.

### Selection pressure analysis

To interpret gene evolutionary trends, the dN/dS ratios (ω = dN/dS) of CDSs in *Vincetoxicum* species were calculated. Each of the 14 *Vincetoxicum* chloroplast genomes was aligned with *Trachelospermum jasminoides* using MAFFT ver. 7.490 (Katoh and Standley 2013). Based on the extracted protein-coding genes, dN/dS calculations were performed using the PAML package (Yang 2007). The codeml module was employed, and dN/dS ratios were calculated using a pairwise model (runmode = − 2, model = 0) in PAML. Nineteen highly conserved genes with no or insignificant variation were not included in Table S6. The final dN/dS values were visualized using R package ggplot2 (Wickham 2016). The dN/dS values were classified by gene functional groups to examine the categories of genes that exhibited evolutionary signals.

### Phylogenetic analysis

For phylogenetic analysis, protein-coding genes from 16 chloroplast genomes were utilized, comprising 14 *Vincetoxicum* species as the ingroup and *Hoya pottsii* and *T. jasminoides* as the outgroup. Except for *V. inamoenum* and *V.* sp., the remaining 14 chloroplast genomes were downloaded from the NCBI database (Table S1). All chloroplast genomes were aligned using MAFFT ver. 7.490 (Katoh and Standley 2013), and 73 protein-coding genes were extracted after alignment, excluding *infA*, *rps12*, and *ycf*. To improve alignment quality, Gblocks ver. 5 (Castresana 2000) was used to eliminate highly variable and poorly aligned regions. The best-fit nucleotide substitution model was determined using jModelTest ver. 2.1.10 (Posada 2008), selecting the GTR + I+G model based on the Akaike Information Criterion (AIC). Maximum Likelihood (ML) phylogenetic trees were constructed in MEGA 11 (Tamura et al. 2021) using the GTR + I + G model and 1,000 bootstrap replicates. Bayesian Inference (BI) analysis was performed using MrBayes ver. 3.2.6 (Huelsenbeck and Ronquist 2001) using the Geneious software plugin, applying the GTR model with four gamma rate categories, and running the Markov Chain Monte Carlo (MCMC) algorithm for 5,000,000 generations with two independent runs of two chains each. Tree sampling was performed every 200 generations, with the first 1,250,000 (25%) generations discarded as burn-ins.

### Divergence time estimation

Divergence time was estimated using BEAST ver. 2.7.7 (Bouckaert et al. 2019), with the analysis setup performed in BEAUti. The analysis was configured using the GTR site model, relaxed log-normal molecular clock, and Yule model as tree priors. The site model parameters were based on results from jModelTest ver. 2.1.10 (Posada 2008). For divergence time calibration, one fossil calibration point and one secondary calibration point were established based on a previous study (Liede-Schumann et al. 2012): [1] the root node, representing the common ancestor of all species, was calibrated with an upper bound of 53.0 million years ago (MYA) and a lower bound of 47.0 MYA based on *Apocynospermum* fossil evidence; [2] the node representing the most recent common ancestor of all *Vincetoxicum* species was calibrated to 18.35 MYA (95% HPD: 11.53–25.09) based on the crown age of the subtribe Tylophorinae. The MCMC analysis was run for 200 million generations, with 10% burn-in and sampling every 10,000 generations. Effective sample size (ESS) values were evaluated using Tracer ver. 1.7.2 (Rambaut et al. 2018), and the resulting trees were summarized and annotated using TreeAnnotator ver. 2.7.7. Tree visualization, including divergence times and 95% highest posterior density (HPD) intervals, was performed using FigTree ver. 1.4.4 (Rambaut 2018).

## Results

### Identification of the chloroplast genome of *V. inamoenum* and *V.* sp.

**Fig. 2:**
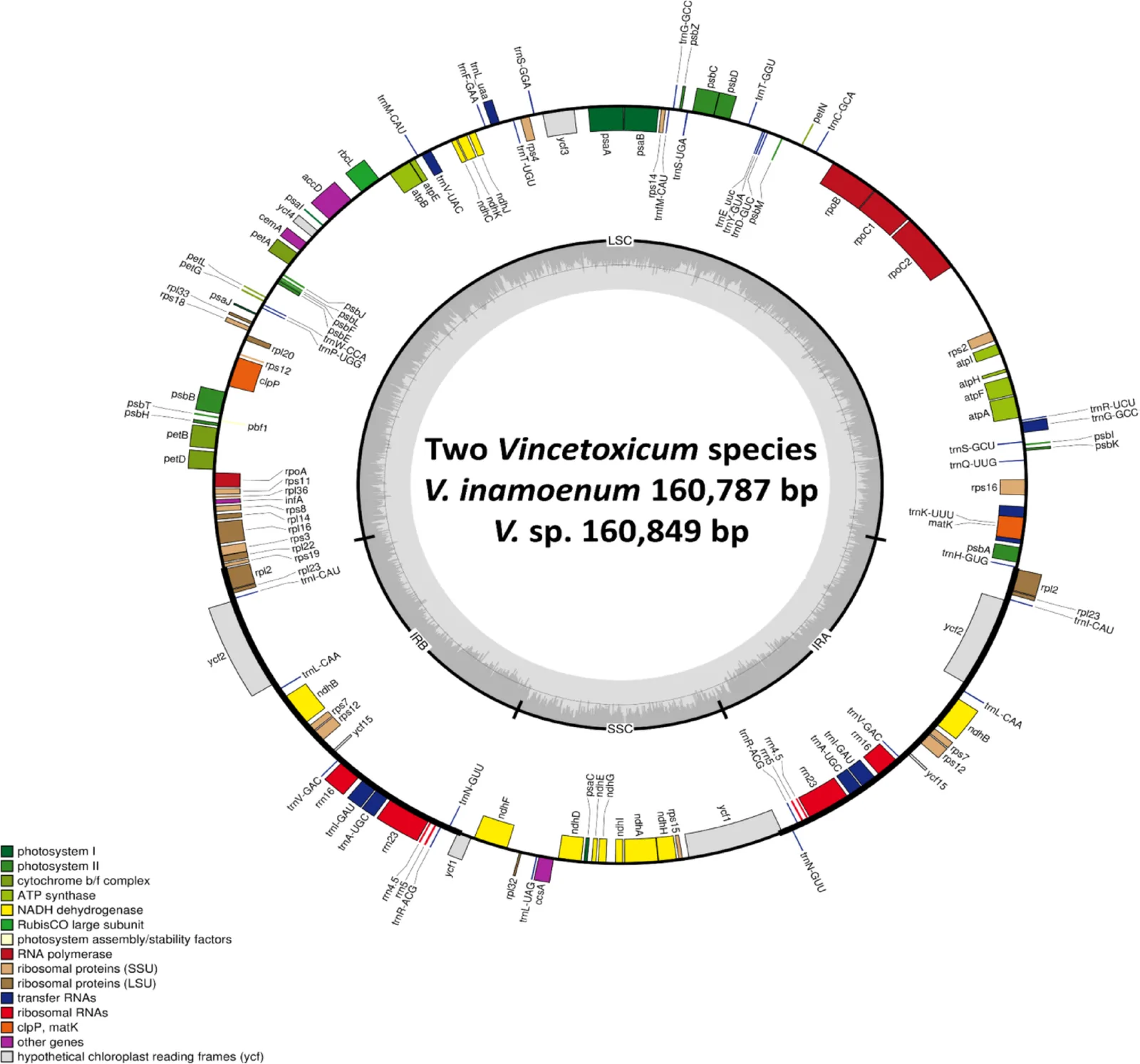
Circular chloroplast genome map of two *Vincetoxicum* species. Genes located inside are transcribed clockwise, whereas those located outside are transcribed counterclockwise. The gray circle at the center of the genome map indicates the GC content across different regions, including the large single-copy (LSC), small single-copy (SSC), and two inverted repeat (IR) regions (IRa and IRb)

We sequenced *V. inamoenum* and *V.* sp., and the genome assembly data as well as raw and trimmed read information are provided in Tables S2 and S3. The total chloroplast genome lengths of *V. inamoenum* and *V.* sp. were 160,787 bp and 160,849 bp, respectively (Table 1). Both chloroplast genomes were assembled in a quadripartite structure consisting of a large single-copy (LSC), a small single-copy (SSC), and two IR regions, IRa and IRb (Fig. 2). The lengths of the LSC, SSC, and IR regions of *V. inamoenum* and *V.* sp. were 91,688 and 91,764 bp; 19,265 and 19,405 bp; and 24,917 and 24,840 bp, respectively. Both chloroplast genomes contained 114 unique genes, including 80 protein-coding, 30 tRNA, and 4 rRNA genes (Table 1). All rRNA genes (*rrn4.5*, *rrn5*, *rrn16*, and *rrn23*) were located in the IR region (Table S4). Both species contained 18 intron-containing genes. Thirteen of these genes were located in the LSC region. Fifteen of these 18 genes had a single intron, while *clpP* and *ycf3* contained two introns, and *rps12* was transspliced gene. *rpl2*, *ndhB*, *trnI-GAU*, and *trnA-UGC* were located in the IR regions, indicating the presence of two copies of these genes (Table S5). To examine codon usage patterns, including the frequencies of 20 amino acids and stop codons, as well as the total number of codons, a codon usage analysis was conducted on 88 protein-coding genes. *V. inamoenum* and *V.* sp. contained 26,654 and 26,642 codons, respectively (Fig. S1a). The amino acids encoded by the largest number of codons were arginine (Arg), leucine (Leu), and serine (Ser), and the most frequently used codons were AGA, UUA, and UCU. Additionally, except for methionine (Met), tryptophan (Trp) was the only amino acid encoded by the single codon, UGG (Fig. S1b). The GC content of *V. inamoenum* and *V.* sp. was 37.7% and 37.8%, respectively (Table 1). The GC content of the main regions of the chloroplast genome was consistent between *V. inamoenum* and *V.* sp., with the IR regions having the highest GC content (43.3%), followed by LSC (36.0%) and SSC (31.7%). Finally, to identify differences between the genomes of *V. inamoenum* and *V.* sp. and those of the 12 previously identified *Vincetoxicum* species, chloroplast genome information for these 12 species was collected from the NCBI database and summarized into corresponding categories (Table 1). No apparent differences in genome length, gene number, or GC content were observed. These results indicate that the *Vincetoxicum* chloroplast genome was conserved.

**Table 1:** Characterization of *Vincetoxicum* chloroplast (cp) genomes

Gene property		Species		
		*Vincetoxicum inamoenum*	*Vincetoxicum sp.*	*Twelve Vincetoxicum*
Total cp genome size (bp)		160,787	160,849	159,666 ~ 161,280
Large single-copy (LSC) region (bp)		91,688	91,764	90,467 ~ 92,084
Inverted repeat (IR) region		24,917	24,840	24,810 ~ 25,021
small single-copy (SSC) region (bp)		19,265	19,405	19,088 ~ 19,428
Protein-coding gene (unique)		88 (80)	88 (80)	88 (80)
tRNA (unique)		37 (30)	37 (30)	37 (30)
rRNA (unique)		8 (4)	8 (4)	8 (4)
GC content (%)	Genome	37.7	37.8	37.7 ~ 37.8
	LSC region	36.0	36.0	36.0 ~ 36.1
	IR region	43.3	43.3	43.2 ~ 43.4
	SSC region	31.7	31.7	31.6 ~ 32.0

### Repeat analysis

**Fig. 3:**
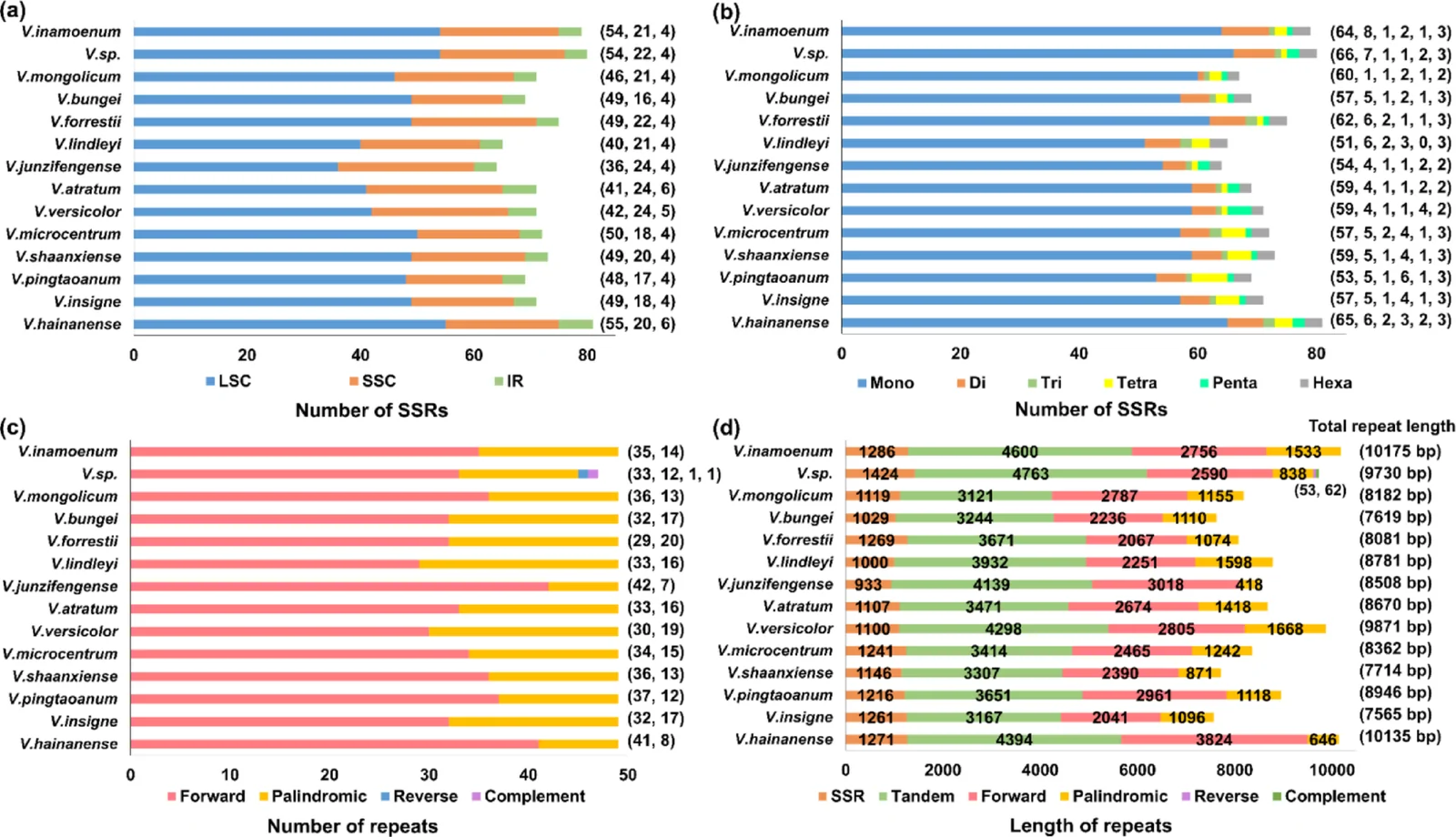
Distribution of repeat sequences of 14 *Vincetoxicum* chloroplast genomes. **a** Distribution of SSRs across chloroplast genomic regions (LSC, SSC, IR regions); **b** type composition and number of SSRs; **c** frequency of repeat sequence types (forward, reverse, palindromic, complement) in *Vincetoxicum* chloroplast genomes; **d** total length of repeat sequences. In (**a**) to (**c**), each number in parentheses indicates the quantified value of each category

To summarize the composition of repetitive sequences in the chloroplast genomes of *V. inamoenum*, *V.* sp., and the 12 NCBI-annotated *Vincetoxicum* species, repeat sequences were analyzed (Fig. 3 and Fig. S2). Among the fourteen *Vincetoxicum* species, total SSR counts ranged from 64 (*V. junzifengense)* to 81 (*V. hainanense)* (Fig. 3a). Regionally, the distribution ranged from 36 to 55 SSRs in the LSC, 17 to 24 in the SSC, and 4 to 6 in the IR, indicating that the highest abundance was in the LSC and the lowest in the IR regions. A total of 79 SSRs in *V. inamoenum* and 80 SSRs in *V.* sp. were identified, differing slightly in the SSC region with 21 and 22 SSRs, respectively.

Regarding SSR motif types in the 14 *Vincetoxicum* species, mono- to hexanucleotide simple sequence repeat (SSR) motifs were detected in 14 Vincetoxicum species. Mononucleotides were the most abundant and ranged from 51 to 66. Simple pentanucleotide SSR motifs were absent in *V. lindleyi*. Slight differences in the number of SSRs between *V. inamoenum* and *V.* sp. were observed, with 64 and 66 mononucleotide SSRs, eight and seven dinucleotide SSRs, two and one tetranucleotide SSRs, and one and two pentanucleotide SSRs, respectively (Fig. 3b).

The total number of tandem repeats of all 14 *Vincetoxicum* species ranged from 62 (*V. bungei*) to 82 (*V.* sp.). Regionally, the LSC contained the highest number of tandem repeats (29–42), followed by the IR regions (16–24), whereas the SSC regions had the fewest tandem repeats (10–19) (Fig. S2a). In total, 76 and 82 tandem repeats were identified in *V. inamoenum* and *V.* sp., respectively, differing in the number of repeats in the SSC region: 13 in *V. inamoenum* and 19 in *V.* sp. We examined the length distribution of tandem repeats and found that tandem repeats of 21–40 bp were the most abundant in all species, whereas repeats of 81–100 bp were the least frequent (Fig. S2b). Among the detected tandem repeats, the longest one, 384 bp (motif of 204 bp with 1.8 copies), was identified in *V. lindleyi*. *V. inamoenum* and *V.* sp. possessed 34 and 33 repeats of 21–40 bp, 21 and 23 repeats of 41–60 bp, 7 and 11 repeats of 61–80 bp, and 6 and 7 repeats of 81–100 bp, respectively, as well as eight repeats longer than 101 bp.

Most *Vincetoxicum* species contained only forward (29–42) and palindromic repeats (7–20), and the number of forward repeats was the highest among all species. *V. inamoenum* contained only forward and palindromic repeats (35 and 14, respectively), whereas *V.* sp. exhibited a wider variety in both the number and type of repeats, including 33 forward repeats, 12 palindromic repeats, one reverse repeat, and one complement repeat, indicating differences in repeat-type composition (Fig. 3c).

The total length of the repeats ranged from 7565 to 10,175 bp, with *V. insigne* having the shortest total repeat length and *V. inamoenum* the longest (Fig. 3d). Among all species, repeat lengths followed the order of tandem repeats (3121–4763 bp) and forward repeats (2041–3824 bp). Regarding SSRs (933–1424 bp) and palindromic repeats (418–1668 bp), *V. inamoenum*, *V. mongolicum*, *V. bungei*, *V. lindleyi*, *V. atratum*, *V. versicolor*, *V. microcentrum* showed shorter SSRs than palindromic repeats, whereas *V.* sp., *V. forrestii*, *V. junzifengense*, *V. shaanxiense*, *V. pingtaoanum*, *V. insigne*, *V. hainanense* exhibited longer SSRs than palindromic repeats. Although the total repeat lengths differed slightly between *V. inamoenum* and *V.* sp., they accounted for approximately 6% of total genome length in both species.

### Comprehensive comparison of *Vincetoxicum* chloroplast genomes

To identify structural variations, including the expansion and contraction of the IR regions, the chloroplast genomes of 14 *Vincetoxicum* species were evaluated. Their genomic architecture was investigated using *C. wilfordii* as a reference (Fig. S3). The total length of the 14 *Vincetoxicum* chloroplast genomes ranged from 159,666 bp to 161,280 bp, whereas *C. wilfordii* measured 161,241 bp. Only *V. hainanense* had a chloroplast genome longer than that of *C. wilfordii.* The IR regions (IRa and IRb) of the 14 species ranged from 24,810 to 25,021 bp, with *C. wilfordii* having the shortest IR region at 24,658 bp. Compared to *C. wilfordii*, each IR region in all *Vincetoxicum* species was longer by 152–363 bp. In all *Vincetoxicum* species, the *ndhF* gene was located in the SSC region, 188–256 bp away from the IRb boundary. *C. wilfordii*, it was positioned 425 bp away from the IRb boundary. The *ycf1* gene was situated at the SSC/IRa boundary, with 5,095–5,545 bp located in the SSC region, and 825–881 bp within the IRa region. The *ycf1* gene of *C. wilfordii* was situated at the SSC/IRa boundary, with 5,653 bp located in the SSC region and 377 bp located within the IRa region. The *trnH-GUG* gene was located in the LSC region and positioned 1–3 bp away from the IRa boundary. The *rps19* gene was located in the LSC region and was positioned 3 bp from the IRb boundary in all species, except *V.* sp., where it was directly adjacent to the LSC/IRb boundary.

Using the mVISTA program with *V. inamoenum* as a reference, we visualized overall genome variation among *Vincetoxicum* species (Fig. S4). Sequence variation was more pronounced in the LSC and SSC regions than the IR regions and was greater in non-coding regions, including *trnH-psbA*, *trnQ-psbK*, *trnC-petN*, *atpH-atpI*, *ndhC-trnV-UAC*,* rbcL-accD*, *accD-psaI*, and *cemA-petA.* In coding regions, variations were detected in only a few genes, including *ycf1*,* accD*, and *rpl2*. A comparison with *V.* sp. also revealed sequence divergence in several regions, including the *rps2-rpoC2* and *accD-psaI* intergenic regions.

**Fig. 4:**
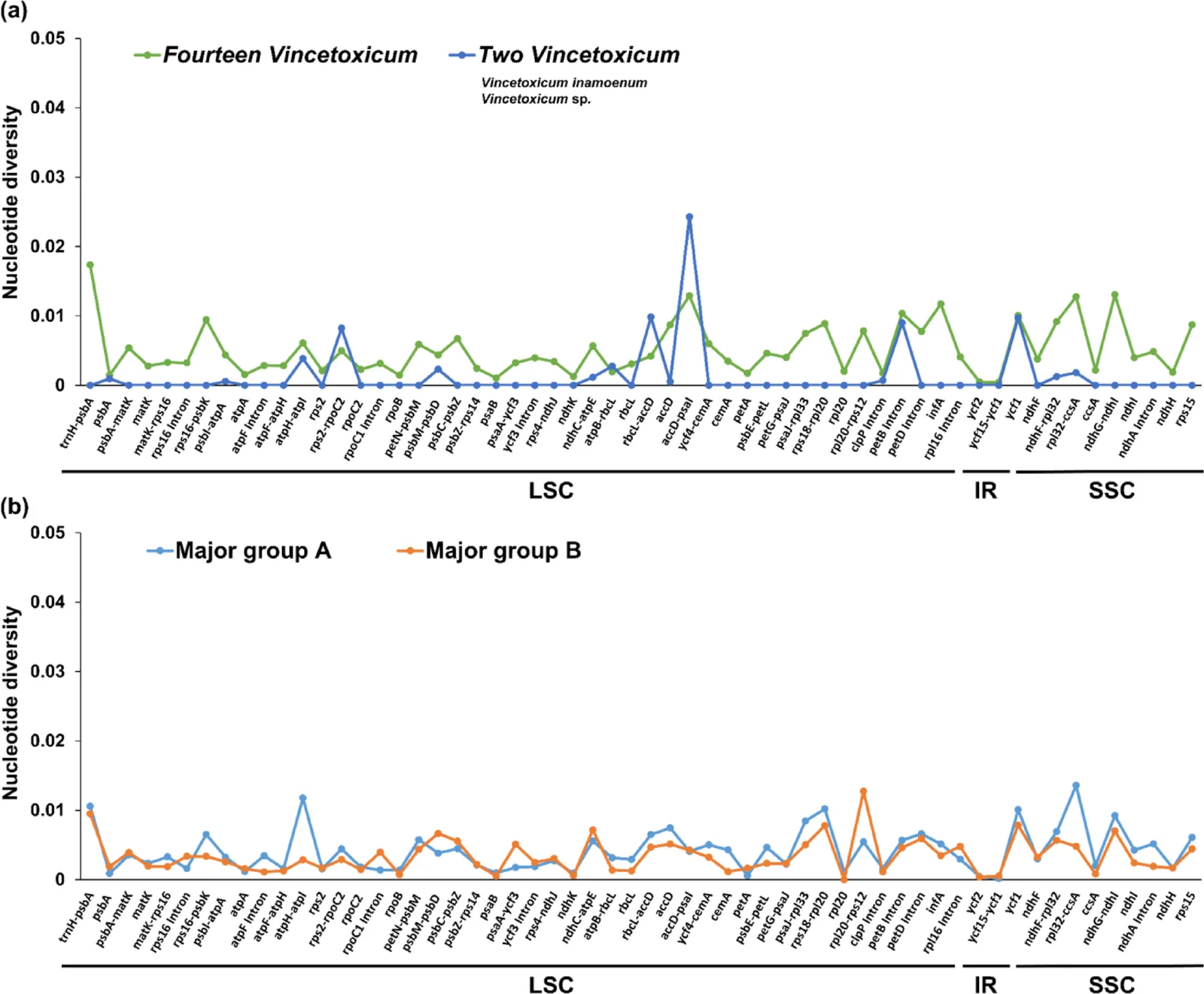
Comparative analysis of nucleotide diversity (Pi) values among 14 *Vincetoxicum* species. **a** Comparison of nucleotide diversity among the 14 *Vincetoxicum* species and separated comparison between the two newly sequenced in this study, *V. inamoenum* and its putative new species, *V.* sp.; **b** comparison of nucleotide diversity within two major groups (Group A and Group B). Major groups are generated according to branch topology in phylogenetic tree. Group A comprises nine species (*V. inamoenum*, *V.* sp., *V. mongolicum*, *V. bungei*, *V. forrestii*, *V. lindleyi*, *V. atratum*, *V. versicolor* and *V. junzifengense*) and Group B comprises five species (*V. microcentrum*, *V. shaanxiense*, *V. pingtaoanum*, *V. insigne* and *V. hainanense*)

Nucleotide diversity (Pi) was calculated using four species groups to assess intragroup variation: all 14 *Vincetoxicum* species, the pair *V. inamoenum* and *V.* sp. (Fig. 4a), the major Group A containing nine species (Group A), and the major group containing five species (Group B) (Fig. 4b). The average Pi value among the 14 *Vincetoxicum* species was 0.00512, and the *trnH-psbA* region showed the highest variation with a Pi value of 0.01737, followed by *ndhG-ndhI* (0.01307), *accD-psaI* (0.01293), and *rpl32-ccsA* (0.01277) (Fig. 4a). Although the coding regions were relatively conserved, high levels of variation were detected in *infA* (0.01172) and *ycf1* (0.01006). In the nine-species group, the highest nucleotide diversity was observed in the *rpl32-ccsA* intergenic region (Pi = 0.0136), whereas the five-species major group, the *rpl20-rps12* intergenic region showed the highest variation (Pi = 0.01276) (Fig. 4b). The average Pi value between *V. inamoenum* and *V.* sp. was 0.00133, which was the lowest among all groups. However, nucleotide differences were detected in *rps2-rpoC2*, *rbcL-accD*, *accD-psaI*, *petB* intron, and *ycf1* gene. Among these, the *accD-psaI* intergenic region displayed the highest nucleotide diversity with a Pi value of 0.02429 (Fig. 4a).

### dN/dS analysis

**Fig. 5:**
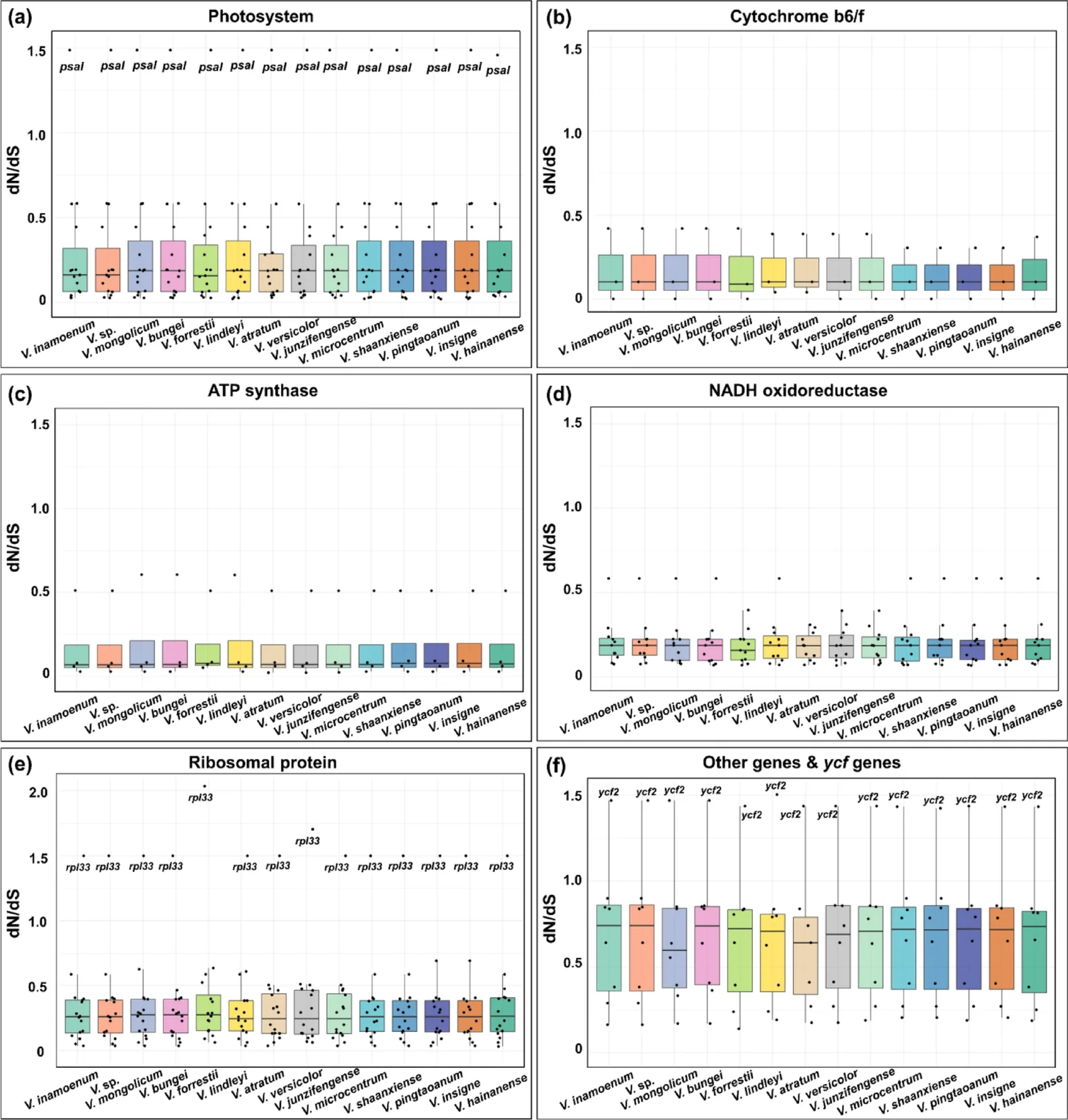
dN/dS values for primary chloroplast-related functional gene groups of 14 *Vincetoxicum* species. **a** Photosystem-related functional gene group; **b** cytochrome b6/f complex-related functional gene group; **c** ATP synthase-related functional gene group; **d** NADH oxidoreductase-related functional gene group; **e** ribosomal protein-related functional gene group; **f** other functional genes, including *accD*, *clpP*, *ycf1*, and *ycf2*

To examine evolutionary trends of protein-coding genes in the 14 *Vincetoxicum* species, we performed a selection pressure analysis by calculating the dN/dS ratio using *T. jasminoides* as a reference. Seventy-seven of the 80 protein-coding genes exhibited ω values ranging from 0 to 0.8981 (ω < 1), indicating purifying selection. Notably, three genes—*psaI* (encoding a photosystem I subunit), *rpl33* (encoding a ribosomal protein), and *ycf2* (an essential gene for cell viability)—showed ω > 1. These results suggest that except for the *ycf2* gene, the two genes associated with photosynthesis (*psaI*) and protein synthesis (*rpl33*) provide potential signals of positive selection (Fig. 5a, e and Table S6).

### Phylogenetic relationships among *Vincetoxicum* species

**Fig. 6:**
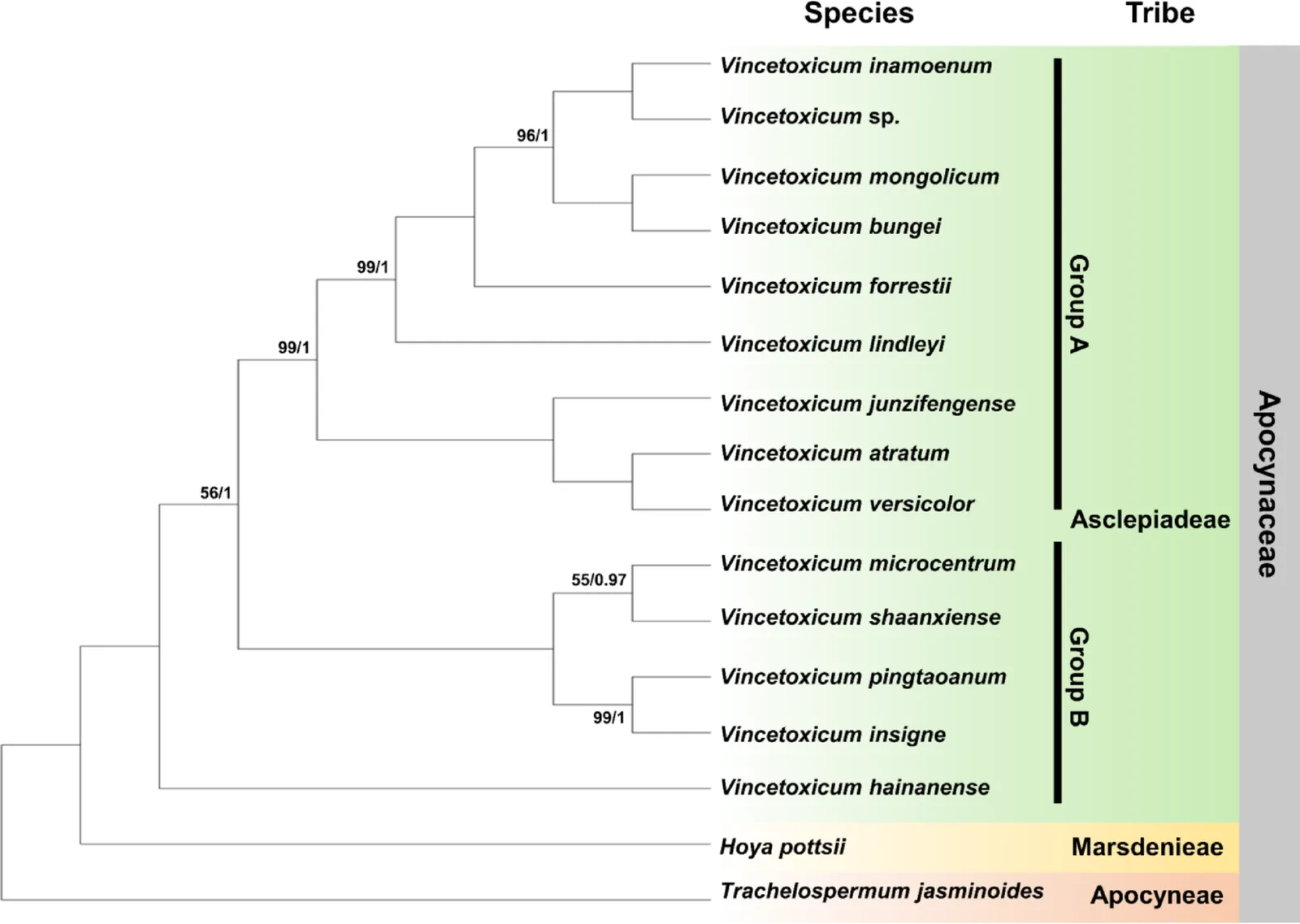
Phylogenetic tree of 14 *Vincetoxicum* species based on a dataset of 73 protein-coding genes. Based on the chloroplast genomes of 14 *Vincetoxicum* species, phylogenetic trees were independently constructed using the maximum likelihood (ML) and Bayesian inference (BI) methods, with *Hoya pottsii* and *Trachelospermum jasminoides* as outgroups, and were merged into a single tree because identical results were obtained. Two values separated by a slash (/) on several nodes indicate the ML bootstrap value (left) and BI posterior probability. Note that the bootstrap values and posterior probabilities are not shown when they are 100 and 1, respectively

To confirm the phylogenetic relationship between *V*. *inamoenum* and *V*. sp. and the previously reported 12 *Vincetoxicum* species, we constructed a phylogenetic tree using 16 species of Apocynaceae. This included 14 *Vincetoxicum* species as the ingroup, whereas *H. pottsii* and *T. jasminoides* were utilized as outgroups, following the methodology described by Jeong et al. (2025) (Fig. 6). Phylogenetic trees were generated using both Maximum Likelihood (ML) and Bayesian Inference (BI) methods, and the resulting topologies were consistent across both approaches, showing strong support for most nodes (ML = 100%, BI = 1.0). Within the ingroup of the constructed tree, two major groups were identified based on the tree topology (Groups A and B). Group A consisted of nine species (*V. inamoenum*, *V.* sp., *V. mongolicum*, *V. bungei*, *V. forrestii*, *V. lindleyi*,* V. atratum*, *V. versicolor*, *V. junzifengense*), whereas Group B consisted of five species (*V. microcentrum*, *V. shaanxiense*, *V. pingtaoanum*, *V. insigne*, *V. hainanense*). The two major groups were divided into three clusters. Cluster 1 (*V. inamoenum*, *V.* sp., *V. mongolicum*, *V. bungei*, *V. forrestii*, *V. lindleyi*) and Cluster 2 (*V. atratum*, *V. versicolor*, *V. junzifengense*) together comprised Group A, forming a closely related lineage. In contrast, in Group B, four of the five species (*V. microcentrum*, *V. shaanxiense*, *V. pingtaoanum*, *V. insigne*) formed Cluster 3, whereas the remaining species, *V. hainanense*, was identified as the basal lineage of the ingroup. In the first cluster, *V. inamoenum* formed a sister group with *V.* sp. (ML = 100%, BI = 1.0), and this branch was strongly supported. Within this cluster, *V. mongolicum* and *V. bungei* were identified as sister species. *V. atratum* and *V. versicolor* formed a sister group that comprised the second cluster. Finally, within the third cluster, *V. microcentrum* and *V. shaanxiense* and *V. pingtaoanum* and *V. insigne*, formed two sister pairs that together comprised Cluster 3.

### *Vincetoxicum* divergence time estimation

**Fig. 7:**
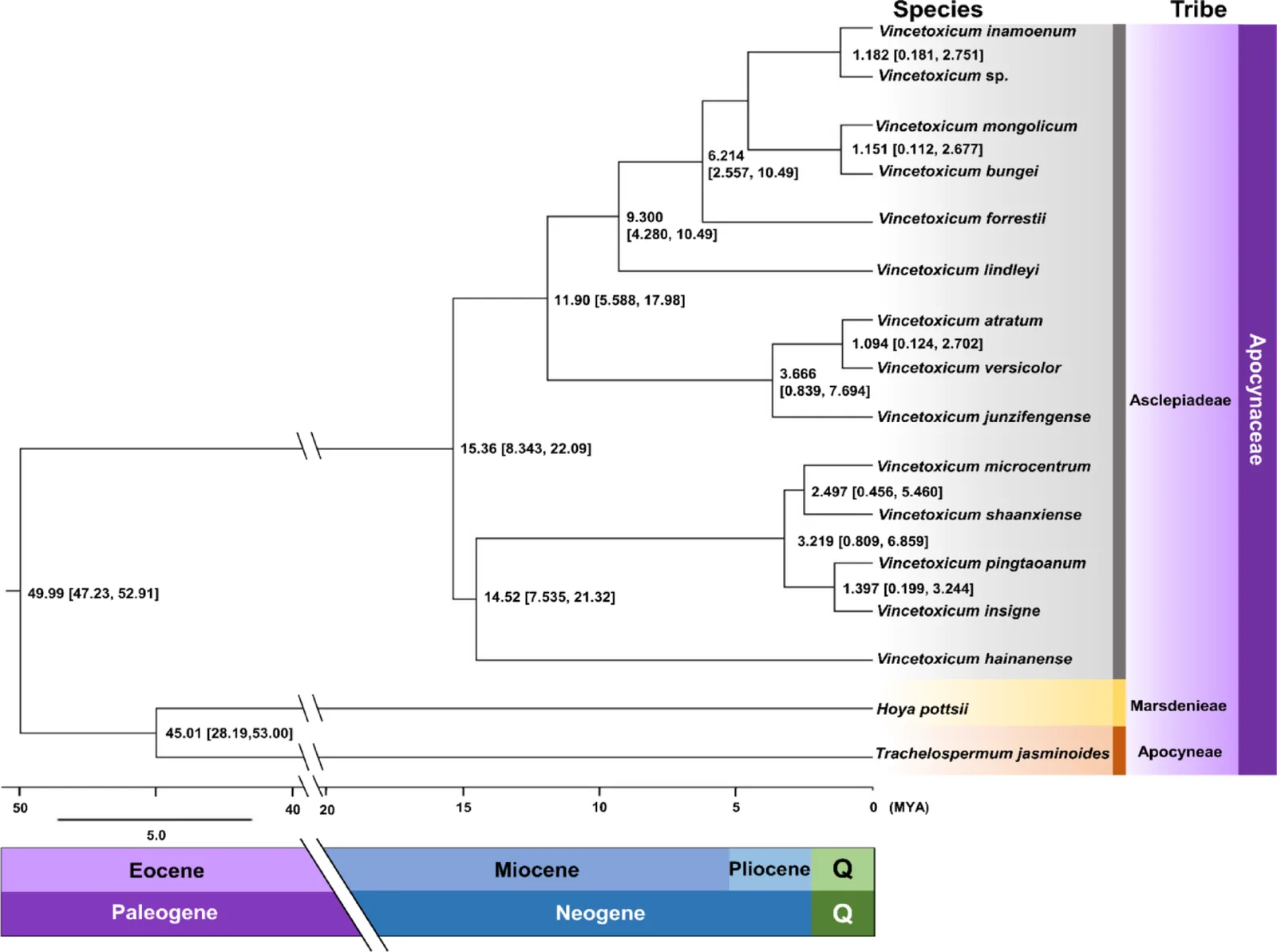
Divergence time estimation of *Vincetoxicum* species. Divergence times and a molecular clock tree were generated using protein-coding gene sequences from 14 *Vincetoxicum* species and two outgroups using the BEAST program, with one fossil calibration point and one secondary calibration point. The root node represented the common ancestor of all species, and the node represents the most recent common ancestor of all *Vincetoxicum* species. Each node was labeled with the estimated divergence time (million years ago) and 95% highest posterior density (HPD) interval: P = Pliocene; Q = Quaternary (Pleistocene + Holocene)

To investigate evolutionary divergence among *Vincetoxicum* species based on phylogenetic relationships, we estimated the divergence times of 14 *Vincetoxicum* species and two outgroup species (16 in total) using 73 protein-coding genes (Fig. 7). The tree generated using BEAST was congruent with the phylogenetic tree obtained from the previous analyses. *T. jasminoides* and *H. pottsii* diverged from the *Vincetoxicum* genus approximately 49.99 MYA (95% highest posterior density [HPD]: 47.23–52.91). This divergence occurred during the Early and Middle Eocene. Divergence of the two major groups, Group A and Group B, identified within the *Vincetoxicum* phylogenetic analysis was estimated to have occurred around 15.36 MYA (95% HPD: 8.343–22.095). According to the phylogenetic tree, *V. hainanense*, identified as the earliest-diverging species within the ingroup, was estimated to have diverged from the species in the third cluster (*V. microcentrum*, *V. shaanxiense*, *V. pingtaoanum*, and *V. insigne*) by approximately 14.523 MYA (95% HPD: 7.535–21.329). The divergence between the first and second clusters was estimated to be 11.903 MYA (95% HPD: 5.588–17.985). Except for *V. forrestii*, *V. lindleyi*, and *V. hainanense*, most *Vincetoxicum* species were estimated to have diverged by approximately 3.666–1.094 MYA (95% HPD: 0.112–7.694), corresponding to the period from the Pliocene to the Pleistocene. Of these, *V.* sp. was estimated to have diverged from *V. inamoenum* approximately 1.182 MYA (95% HPD, 0.181–2.751).

## Discussion

### Characterization of the chloroplast genome and genetic variations in *Vincetoxicum*

In the present study, we assembled the chloroplast genomes of two *Vincetoxicum* species, *V. inamoenum* and *V.* sp., for which chloroplast genome sequences have not yet been reported in public databases. The lengths of the complete chloroplast genomes of both species fell within the typical genome size range of higher plant chloroplast genomes, from 120 to 180 kb (Xu et al. 2021). The genome structure also exhibited a typical quadripartite organization comprising an LSC region, an SSC region, and two IR regions, as observed in other angiosperm chloroplast genomes (Wang and Lanfear 2019) (Fig. 2). All *Vincetoxicum* species exhibited the highest GC content in the IR region (Table 1), consistent with typical chloroplast genomes, suggesting that the GC-rich IR region contributes to structural stability and lower sequence variation in the genome of *Vincetoxicum* species (Zhang et al. 2011; Zhong et al. 2025). Previous studies reported that *ycf15* is either lost or exists as a pseudogene in several angiosperms (Raubeson et al. 2007; Shi et al. 2013). In this study, all *Vincetoxicum* chloroplast genomes contained the *ycf15* gene, and their sequences were identical (Table S4). Additional studies are needed to determine whether these *ycf15* sequences represent functional genes, and if so, to clarify their possible roles within chloroplasts. Approximately 69% of the codons in *V. inamoenum* and *V.* sp. ended in A or U nucleotides. This trend has also been observed in other higher plants (Ye et al. 2024) and may be attributed to the A/T-rich structure of the chloroplast genome (Abdullah et al. 2020; Mehmood et al. 2020). In addition, most A/U-ending codons tended to have RSCU values exceeding 1, as has been reported for many plant species (Saina et al. 2018; Gao et al. 2024; Park et al. 2024).

### Comparative genomic insights into chloroplast genome of *Vincetoxicum* species

Repeated sequences are involved in genome rearrangement and recombination, which, in turn, contribute to evolutionary changes (Nie et al. 2012). SSRs are widely distributed across most plant genomes, and are commonly used to evaluate genetic diversity (Sonah et al. 2011; Cho et al. 2024; Liu et al. 2025). SSRs in all *Vincetoxicum* species were most frequently distributed in the LSC region, as has been observed in several other plant species (Xu et al. 2023; Yu et al. 2025; Jeong et al. 2025) (Fig. 3a). SSR motif-type analysis revealed that mononucleotide repeats were predominantly distributed across all *Vincetoxicum* species, with most SSR motifs composed of A/T bases, consistent with the previous assumption that chloroplast genome SSRs are mainly composed of short poly-A and poly-T repeats (Li et al. 2018; Duan et al. 2022; Fig. 3b). Previous studies have suggested that repeats longer than 20 bp facilitate genome rearrangement in Geraniales (Weng et al. 2014). Since all tandem repeats identified in this study were at least 21 bp in length, these repeats may also have contributed to genome rearrangements (Fig. S2b). These repeat sequences may also contribute to variations in the chloroplast genome size (Guo et al. 2021; Kim et al. 2025). The total length of the repeat sequences varied among *Vincetoxicum* species, ranging from 7565 to 10,175 bp (Fig. 3d). In the present study, species with relatively long repeats (*V. inamoenum*, *V.* sp., and *V. hainanense*) tended to have larger chloroplast genomes, although this pattern was not observed in *V. versicolor*. In these repeat sequences, the observed differences in both the number and length of repeats between *V. inamoenum* and *V.* sp. indicated potential genetic differentiation. In addition, the overall pattern of Pi values among the 14 species was consistent with the mVISTA results, showing high variation in non-coding regions and relatively elevated values in coding regions of *accD*, *infA*, and *ycf1*. In particular, the *trnH-psbA* region exhibited the highest Pi value (Fig. 4a). Although little divergence was expected between each of the two major groups, each group nonetheless exhibited distinct high-divergence regions (Group A: *atpH-atpI*, *rpl32-ccsA*; Group B: *rpl20-rps12*). Integration of the mVISTA and Pi results between *V. inamoenum* and *V.* sp. suggested genetic differences, with the most pronounced variation observed in the *accD-psaI* region (Pi = 0.02429). Previous studies have employed regions such as *trnH-psbA* (across 14 species) and *accD-psaI* (between *V. inamoenum* and *V.* sp.) as DNA barcoding markers for species discrimination (Kress et al. 2009; Li et al. 2025). Therefore, these regions may serve as potential DNA barcoding markers. However, further validation using broader taxon sampling or population-level datasets is required before these regions can be used as practical DNA barcodes.

### Selection pressure analysis

dN/dS analysis provides important insights into the adaptive evolution of protein-coding genes (Kryazhimskiy and Plotkin 2008). The dN/dS ratio (ω) can reflect three types of natural selection: purifying selection (ω < 1), neutral selection (ω = 1), and positive selection (ω > 1) (Yang and Nielsen 2002). Seventy-seven of the 80 protein-coding genes were under purifying selection, suggesting that *Vincetoxicum* evolved conservatively under strong purifying selection, reflecting a slow rate of molecular evolution (Fig. 5 and Table S6). However, *psaI*, *rpl33*, and *ycf2* are potential signals for positive selection. Positive selection of *ycf2* has been repeatedly reported across diverse plant lineages (Chen et al. 2020; Wu et al. 2020), and *psaI* and *rpl33* genes have also been reported to be under positive selection (Tian et al. 2021; Zhou et al. 2021; Li and Wei 2022; Yu et al. 2022; Zhu et al. 2024). This suggests that amino acid substitutions occurred as an adaptation to the environment (Huang et al. 2020). Given that these positive selection signals were observed in all analyzed species, *Vincetoxicum* may have originated from a common ancestor. Thus, because of its divergence from *T. jasminoides*, *Vincetoxicum* may have undergone amino acid substitutions related to environmental adaptation since its divergence from *T. jasminoides*.

### Phylogenetic analysis of *Vincetoxicum* species

Until recently, phylogenetic analyses of *Vincetoxicum* using molecular data have been continuously conducted. Using the complete chloroplast CDS data from 14 species, we reconstructed the phylogenetic relationships of *Vincetoxicum* (Fig. 6). Two major groups (Groups A and B) were strongly supported in the Bayesian tree (posterior probability = 1), but showed relatively low bootstrap support in the ML tree (ML = 56). Therefore, these two major groups should be interpreted with caution considering the methodological differences between the two approaches. *Vincetoxicum* has long been considered a taxonomically problematic genus (Kidyoo et al. 2024). Previous molecular phylogenetic studies have shown that *Vincetoxicum*, *Tylophora*, and *Biondia* do not form a monophyletic group, and therefore proposed that these genera be merged into *Vincetoxicum sensu lato* (Yamashiro et al. 2004; Liede et al. 2012). These studies further suggest that the monotypic genus *Merrillanthus* may also need to be incorporated, based on its morphological affinity (Liede et al. 2012). Subsequent taxonomic revisions formally transferred species of *Tylophora*, *Biondia*, and *Merrillanthus* into *Vincetoxicum* (Liede et al. 2018; Zhang et al. 2022a, b). In our study, *V. lindleyi*, formerly classified as *Tylophora hirsuta*, was recovered within Group A together with other *Vincetoxicum* species, supporting the previously reported non-monophyly of these lineages. Group B consisted of five species, four of which were previously assigned to *Biondia* or *Merrillanthus*. *V. microcentrum* ( *B. microcentra*), *V. shaanxiense* ( *B. chinensis*), *V. insigne* ( *B. insignis*), and the recently described *V. pingtaoanum* formed a cluster, and *V. hainanense* (*M. hainanensis*) was recovered as the basal lineage of *Vincetoxicum*. The relationship of *V. pingtaoanum* is consistent with its morphological similarity to former *Biondia* species and agrees with unpublished preliminary phylogenetic analyses in the original species description, which also identified it as the closest to the *Biondia* lineage (e.g., *V. insigne*,* V. microcentrum*) (Zhang et al. 2022a, b). These patterns indicate that the previously inferred non-monophyly of several constituent lineages within *Vincetoxicum* s.l.—as suggested by morphological data, nuclear ribosomal DNA, and limited chloroplast DNA regions—is corroborated by the complete chloroplast data. However, the separation of species formerly treated as *Tylophora* and *Biondia* into distinct clades was partially inconsistent with the results of previous studies (Liede et al. 2012). This discrepancy may be attributed to the limited availability of the complete chloroplast genome data for this genus. The newly assembled species, *V. inamoenum* and *V.* sp., were recovered as sister groups (ML = 100, BI = 1). Additionally, our analysis included *V. pingtaoanum* and *V. insigne*, resulting in two sister pairs: *V. microcentrum* and *V. shaanxiense*, and *V. pingtaoanum* and *V. insigne*, thereby restructuring the sister-group relationships suggested by previous research (Ren et al. 2025). This separation leads to the formation of two new sister groups. Our results are congruent with those of other phylogenetic trees of *Vincetoxicum* based on complete chloroplast genomes (Yu et al. 2021; Liu et al. 2023; Ren et al. 2025), whereas discrepancies were found in some analyses using only limited regions. Previous phylogenetic analyses using nuclear ribosomal DNA regions (internal and external transcribed spacers) and limited chloroplast regions have revealed several topological conflicts compared to our results. Yamashiro et al. (2004) suggested that *V. inamoenum* formed an independent clade separated from *V. bungei* (previously referred to as *V. pycnostelma* Kitag) and *V. atratum*, whereas in our chloroplast-based tree, *V. inamoenum* and *V. bungei* were placed within the same cluster.

Kidyoo et al. (2024) reported that *V. inamoenum*, *V. atratum*, and *V. versicolor* formed clusters; however, our results showed that *V. inamoenum*, *V. mongolicum*, and *V. forrestii* formed cohesive clusters. These examples indicate that phylogenetic trees of *Vincetoxicum* constructed from limited genomic regions can show conflicting signals compared to those inferred from complete genome data. Topological conflicts with nuclear ribosomal DNA may be attributed to chloroplast capture (Liu et al. 2020). However, previous studies have reported phylogenetic discrepancies between the *V. bungei* chloroplast genome used in the present study (OK271107.1) and a newly deposited *V. bungei* chloroplast genome (PV747498) (Han et al. 2026). The authors suggested that such differences might be attributable to either the misidentification of related sequences in the NCBI database or the presence of previously unrecognized cryptic species divergence. Therefore, the phylogenetic topological conflicts observed in the present study may have arisen from these factors. Many previous studies have demonstrated that phylogenetic analyses using whole-chloroplast datasets provide higher support values and contribute to a clearer resolution of phylogenetic relationships (Feng et al. 2020; Kyalo et al. 2020; Xiong et al. 2023). Therefore, we constructed a high-resolution phylogenetic tree using protein-coding genes from complete cp genomes, which provided high support values. However, limitations remain, such as nodes with low bootstrap support. Thus, additional analyses using nuclear and mitochondrial data, as well as the assembly of additional *Vincetoxicum* chloroplast genomes, are necessary to further clarify the taxonomy and phylogenetic relationships within the genus.

### Divergence time and evolutionary dynamics of *Vincetoxicum*

To interpret the evolutionary interspecific relationships of *Vincetoxicum*, we estimated the divergence time using complete chloroplast genome CDS data. Fossil calibration is generally performed to estimate divergence times (Feldberg et al. 2013; Barba-Montoya et al. 2017), but direct calibration for the genus *Vincetoxicum* is difficult due to the lack of fossil records, even at the subtribe level of Tylophorinae, to which *Vincetoxicum* belongs (Liede-Schumann et al. 2012). For this reason, *Apocynospermum* (47.0–53.0 MYA), the oldest fossil encompassing the common ancestor of all species included in our analysis, was used to calibrate the root node. Subsequently, the crown age estimate of subtribe Tylophorinae (mean: 18.35 MYA, 95% HPD: 11.53–25.09), to which *Vincetoxicum* belongs, presented in a previous study (Liede-Schumann et al. 2012) based on five chloroplast regions (*trnG*,* trnL*,* trnL-F*,* trnT-L*,* psbA-trnH*) calibrated with the aforementioned fossil, was adopted as a secondary calibration point. However, this calibration point does not represent the crown age of the lineage itself but that of the broader taxonomic group Tylophorinae. Therefore, applying this value to the present analysis may lead to an overestimation of the actual divergence time. The reference species used in the dN/dS analysis, *T. jasminoides*, diverged from *Vincetoxicum* during the Eocene (Fig. 7). The integration of the dN/dS results suggests that an event triggering positive selection may have occurred after that period. Although previous studies estimated the divergence between *Vincetoxicum* and *T. jasminoides* at 34.57 MYA (95% HPD: 28.05–40.36), our results suggest an even earlier divergence (Zhang et al. 2024). This discrepancy may be attributed to differences in sample size and amount of molecular data used. However, *V. mongolicum* and *V. forrestii* were estimated to have diverged at 5.57 MYA (95% HPD: 1.71–13.19), which, although earlier than our results, falls within a similar range, thereby supporting our results (Zhang et al. 2024). In the present study, the genus *Vincetoxicum* diverged into two major groups, 15.36 MYA (95% HPD: 8.343–22.095), followed by subsequent diversification. In addition, in Major Group A, two clusters diverged at 11.903 MYA (95% HPD: 5.588–17.985), and in the third cluster, *V. hainanense* split from the remaining species at 14.523 MYA (95% HPD: 7.535–21.329). Although these early divergences all occurred during the Miocene, most of the currently recognized distinct species appear to have diversified much more recently, around 1–3 MYA. This suggests that major diversification events within the genus took place in the recent past, accompanied by a rapid accumulation of nucleotide substitutions. The divergence time between the newly assembled *V. inamoenum* and *V*. sp. was estimated to be relatively recent, at 1.182 MYA (95% HPD: 0.181–2.751), indicating evolutionary differentiation. These molecular dates provide important insights for further studies of the essential medicinal plant genus *Vincetoxicum*.

## Conclusions

In this study, we assembled the chloroplast genomes of a putatively new species, *V.* sp., and its closely related species, *V. inamoenum*. We examined 14 *Vincetoxicum* chloroplast genomes, including 12 with previously published data. Sequence variation was predominantly observed in non-coding regions but was also detected in coding regions such as *accD*,* infA*, and *ycf1*. IR expansion and positive selection were identified by comparison with closely related species. The high-resolution phylogenetic tree revealed two major groups and three distinct clusters. We also demonstrated genetic divergence between the two species by identifying differences in repeat sequences, sequence variations, and genome structural variations. Phylogenetic analysis with divergence time estimation revealed that *V. inamoenum* and *V.* sp. formed a sister group and diverged approximately 1.182 MYA (95% HPD: 0.181–2.751). Taken together, our results suggest that there is genetic divergence between *V. inamoenum* and *V.* sp., indicating that *V.* sp. may represent a potential new species and enhance our understanding of genetic diversity and evolutionary patterns within the taxonomically complex genus *Vincetoxicum*.

## Supplementary Information

Below is the link to the electronic supplementary material.

Supplementary Material 1

Supplementary Material 2

## Data Availability

The chloroplast genome sequences of *V. inamoenum* and *V.* sp. newly assembled in this study have been deposited in the NCBI database (https://www.ncbi.nlm.nih.gov/) under the accession numbers PX975500 and PX975501. All cp genome data used in this study are publicly available from the NCBI for Biotechnology Information database.
