# Supplementary material for: Complete chloroplast-based analysis reveals the emergence of candidate new species and revised divergence time in Vincetoxicum
Source: Genes Genomics. 2026 Sep 28;48(11):1433–51. doi: 10.1007/s13258-026-01808-1 (PMC13719837; doi:10.1007/s13258-026-01808-1)
Supplement: Supplementary file 1 [file 13258_2026_1808_MOESM1_ESM.docx]

**Supplementary Information**

**Complete chloroplast-based analysis reveals the emergence of candidate new species and revised divergence time in *Vincetoxicum***

Bumjin Jang^1†^, Byung Jun Jin^2†^, Yun Ji Park^2^, Sumin Jeong^1^, Yeseul Kim^1^, Bo-Mi Nam^3*^, and Inkyu Park^1,2*^

^1^ Department of Biology, Changwon National University, Changwon 51140, Republic of Korea

^2^ Global Advanced Institute of Nano Technology, Changwon National University, Changwon 51140, Republic of Korea

^3^ Division of Botany, Honam National Institute of Biological Resources, Mokpo 58762, Republic of Korea

*Corresponding authors

Dr. Bo-Mi Nam

Division of Botany, Honam National Institute of Biological Resources, Mokpo, 58762, Republic of Korea

E-mail: bmnam@hnibr.re.kr

Dr. Inkyu Park

Department of Biology, Changwon National University, Changwon 51140, Republic of Korea

Tel: +82-55-213-3455

Fax: +82-55-213-3459

E-mail: pik6885@cwnu.ac.kr

† These authors contributed equally to this work.

Figure S1. Codon usage patterns and relative synonymous codon usage (RSCU) values of the 20 amino acids and stop codons of 88 protein-coding genes in *V. inamoenum* and *V.* sp.

(a) Number of codons encoding each amino acid and stop codons in the analyzed protein-coding genes. For better understanding of the graph, the number of codons is displayed at the top of each bar. (b) RSCU values of *V. inamoenum* and *V.* sp. The colored squares representing specific triplet codons were arranged below each amino acid to indicate which codons were used to encode the 88 analyzed protein-coding genes. The bar graph displays the RSCU for each amino acid

 **Figure S2.** Tandem repeat sequence distribution and length in *Vincetoxicum.*

(a) Distribution of tandem repeats in the LSC, SSC, and IR regions. (b) Length-based distribution of tandem repeats identified in *Vincetoxicum* species

**Figure S3** The contraction and expansion of inverted repeat (IR) regions and the boundaries between LSC, SSC, and IR regions were compared among *Vincetoxicum* species.

The length variation of each genomic region was determined by identifying the positions of several genes located near the boundaries, such as *rps19, rpl2, ndhF, ycf1,* and *trnH*. Nucleotide lengths (bp) shown in parentheses indicate the length of each genomic region, and others, labeled on one or both ends of the gene, indicate the distance from the adjacent boundary

**Figure S4** Alignment and comparison of chloroplast genomes across 14 *Vincetoxicum* species using the mVISTA program.

Sequence differences are indicated by white highlights and gray arrows represent the direction of transcription. Purple bars represent coding exons, pink bars indicate conserved non-coding regions, and green bars denote the IR (inverted repeat) regions

**Supplementary Table S1.** Voucher specimen information for the chloroplast genomes used in this study.

| **No.** | **Tribe** | **Taxon** | **GenBank accession number** |
| --- | --- | --- | --- |
| 1 |  | *Vincetoxicum inamoenum* | PX975500 |
| 2 |  | *Vincetoxicum* sp. | PX975501 |
| 3 |  | *Vincetoxicum mongolicum* | NC_065505 |
| 4 |  | *Vincetoxicum bungei* | OK271107 |
| 5 |  | *Vincetoxicum forrestii* | NC_060305 |
| 6 |  | *Vincetoxicum lindleyi* | NC_069945 |
| 7 | Asclepiadeae | *Vincetoxicum atratum* | NC_085545 |
| 8 |  | *Vincetoxicum versicolor* | NC_052877 |
| 9 |  | *Vincetoxicum junzifengense* | NC_062962 |
| 10 |  | *Vincetoxicum microcentrum* | NC_082943 |
| 11 |  | *Vincetoxicum shaanxiense* | NC_046785 |
| 12 |  | *Vincetoxicum pingtaoanum* | NC_067062 |
| 13 |  | *Vincetoxicum insigne* | NC_042760 |
| 14 |  | *Vincetoxicum hainanense* | NC_051946 |
| 15 | Marsdenieae | *Hoya pottsii* | NC_042246 |
| 16 | Apocyneae | *Trachelospermum jasminoides* | NC_082280 |

**Supplementary Table S2.** Genome assembly data of two *Vincetoxicum* species.

| **Scientific name** | **Aligned reads (#)** | **Coverage (x)** | **Chloroplast genome length** |
| --- | --- | --- | --- |
| *V. inamoenum* | 756,609 | 1,131.6 | 160,787 bp |
| *V.* sp. | 1,562,652 | 2,321.7 | 160,849 bp |

**Supplementary Table S3.** Raw and trimmed read data of two *Vincetoxicum* species.

| **Scientific name** | **Input reads** | **Trimmed reads** | **Raw bases** | **Trimmed bases** |
| --- | --- | --- | --- | --- |
| *V. inamoenum* | 11,467,716 | 11,330,148 | 3,451,782,516 | 2,624,198,511 |
| *V.* sp. | 11,966,820 | 11,905,445 | 3,602,012,820 | 2,779,248,540 |

**Supplementary Table S4.** Classification of functional gene categories in the two *Vincetoxicum* chloroplast genomes: *V. inamoenum* and *V.* sp.

| **Group of genes** | **Name of genes** |
| --- | --- |
| Photosystem I | *psaA, B, C, I, J, ycf3* ***^2)^****, ycf4* |
| Photosystem II | *psbA, B, C, D, E, F, H, I, J, K, L, M, T, Z, pbf1(psbN),* |
| Cytochrome b6/f | *petA, B* ***^1)^****, D* ***^1)^****, G, L, N* |
| ATP synthase | *atpA, B, E, F* ***^1)^****, H, I* |
| RubisCO large subunit | *rbcL* |
| NADH oxidoreductase | *ndhA* ***^1)^****, B* ***^1),3)^****, C, D, E, F, G, H, I, J ,K* |
| Large subunit ribosomal proteins | *rpl2* ***^3)^****, 14, 16* ***^1)^****, 20, 22, 23* ***^3)^****, 32, 33, 36* |
| Small subunit ribosomal proteins | *rps2, 3, 4, 7* ***^3)^****, 8, 11, 12* ***^2),3),4)^****, 14, 15, 16* ***^1)^****, 18, 19* |
| RNA polymerase | *rpoA, B, C1* ***^1)^****, C2* |
| Unknown function proteins coding-genes | *ycf1* ***^3)^****, ycf2* ***^3)^****, ycf15* ***^3)^*** |
| Other genes | *accD, ccsA, cemA, clpP, infA, matK* |
| Ribosomal RNAs | *rrn4.5* ***^3)^****, rrn5* ***^3)^****, rrn16* ***^3)^****, rrn23* ***^3)^*** |
| Transfer RNAs | *trnA-UGC* ***^1),3^****^)^, trnC-GCA, trnD-GUC, trnE-UUC, trnF-GAA,*  *trnfM-CAU, trnG-GCC, trnG-UCC****^1)^****, trnH-GUG, trnI-CAU* ***^3)^****,*  *trnI-GAU* ***^1),3)^****, trnK-UUU* ***^1)^****,* *trnL-CAA* ***^3)^****, trnL-UAA* ***^1)^****,*  *trnL-UAG, trnM-CAU, trnN-GUU* ***^3^****^)^, trnP-UGG, trnQ-UUG, trnR-ACG* ***^3)^****, trnR-UCU, trnS-GCU, trnS-GGA, trnS-UGA, trnT-GGU, trnT-UGU, trnV-GAC* ***^3)^****, trnV-UAC* ***^1)^****, trnW-CCA,*  *trnY-GUA* |

1) Gene containing a single intron, 2) Gene containing two introns, 3) Two gene copies in IRs, 4) Trans-splicing gene

**Supplementary Table S5.** Identification of intron-containing genes and the lengths of exons and introns in the two *Vincetoxicum* species: *V. inamoenum* and *V.* sp.

| No. | Gene | Region | Exon I | Intron I | Exon II | Intron II | Exon III |
| --- | --- | --- | --- | --- | --- | --- | --- |
| 1 | *trnK-UUU* | LSC | 37 | 2486**^1)^**, 2485**^2)^** | 35 |  |  |
| 2 | *rps16* | LSC | 226**^1)^**, 227**^2)^** | 830 | 41**^1)^**, 40**^2)^** |  |  |
| 3 | *trnG-UCC* | LSC | 23 | 679 | 37 |  |  |
| 4 | *atpF* | LSC | 144 | 713**^1)^**, 706**^2)^** | 411 |  |  |
| 5 | *rpoC1* | LSC | 451 | 785 | 1610 |  |  |
| 6 | *ycf3* | LSC | 124 | 725 | 228 | 799 | 155 |
| 7 | *trnL-UAA* | LSC | 36**^1)^**, 37**^2)^** | 500**^1)^**, 497**^2)^** | 50 |  |  |
| 8 | *trnV-UAC* | LSC | 38 | 590 | 37 |  |  |
| 9 | *rps12* | LSC | 114 |  | 232 |  | 35 |
| 10 | *clpP* | LSC | 69 | 778**^1)^**, 777**^2)^** | 291 | 650 | 273 |
| 11 | *petB* | LSC | 6 | 777**^1)^**, 778**^2)^** | 642 |  |  |
| 12 | *petD* | LSC | 8 | 733 | 475 |  |  |
| 13 | *rpl16* | LSC | 9 | 1117**^1)^**, 1125**^2)^** | 399 |  |  |
| 14 | *rpl2* | IR | 391 | 639 | 436**^1)^**, 434**^2)^** |  |  |
| 15 | *ndhB* | IR | 777 | 684 | 756 |  |  |
| 16 | *trnI-GAU* | IR | 42 | 943 | 35 |  |  |
| 17 | *trnA-UGC* | IR | 38 | 943 | 35 |  |  |
| 18 | *ndhA* | SSC | 553 | 1060**^1)^**, 1051**^2)^** | 545 |  |  |

1) *Vincetoxicum* *inamoenum*, 2) *Vincetoxicum* sp. * *rps12* gene is trans-splicing gene.
